# Outcome of acute ischemic stroke after intra-arterial thrombolysis: A study from India

**Published:** 2016-10-07

**Authors:** Jaydip Ray Chaudhuri, Randhir Kumar, Matapathi Umamahesh, Kandadai Rukmini Mridula, Suvarnal Alladi, Srinivasarao Bandaru

**Affiliations:** 1 Department of Neurology, Yashoda Hospital, Hyderabad, India; 2 Department of Neurosurgery, Yashoda Hospital, Hyderabad, India; 3 Department of Radiology, Yashoda Hospital, Hyderabad, India; 4 Department of Neurology, Nizam’s Institute of Medical Sciences, Hyderabad, India; 5 Department of Neurology, National Institute of Mental Health and Neurosciences, Bangalore, India; 6 Department of Neurology AND Department of Research, Yashoda Hospital, Hyderabad, India

**Keywords:** Acute, Ischemia, Stroke, Thrombolytic Therapy, Outcome

## Abstract

**Background:** Intravenous recombinant tissue plasminogen activator (rt-PA) is the currently standard treatment of acute ischemic stroke within 4.5 hours of the onset of stroke. Recent studies have looked at the benefits of administration of intra-arterial (IA) rt-PA within 8 hours onset of symptoms. Our objective was to assess the outcome of stroke after administration of IA rt-PA in patients with acute ischemic stroke.

**Methods:** We recruited 10 consecutive acute ischemic stroke patients with onset of stroke from 4.5 hours to 6.5 hours. The present study was conducted at Yashoda Hospital, Hyderabad, India, between January 2008 and December 2013. All patients underwent stroke subtyping and were administered rt-PA. We measured the thrombolysis in cerebral infarction (TICI) score after thrombolysis and functional outcomes at time of admission, after 24 hours, 30, 60, and 90 days. A good outcome was defined as modified Rankin Scale (mRS) ≤ 2 after 90 days.

**Results:** Out of 10 patients 9 were men, mean age 56.3 ± 1.8 years and age range from 35-68 years. On stroke subtyping, 6 (60%) patients had large artery atherosclerosis, 3 (30%) had a stroke of indeterminate etiology and 1 (10%) had a stroke of other etiologies. Mean time of recanalization was 6.2 ± 0.5 hours, 7 (70%) patients showed major neurological improvement with a mRS score of ≤ 2 at 90 days and one patient was lost to follow-up.

**Conclusion:** Our study established good outcome at 90 days after administration of IA thrombolysis rt-PA in acute ischemic stroke.

## Introduction

In spite of the wide availability of recombinant tissue plasminogen activator (rt-PA) for the treatment of acute ischemic stroke, stroke still remains the third leading cause of death in developed and developing countries^1^^,^^2^ and around 10% of patients die within 30 days.^3^ The rt-PA is the globally approved drug for treatment of acute ischemic stroke.^4^ It has to be administered intravenously (IV) within 3 to 4.5 hours of the onset of stroke.^4^^,^^5^ Alternatively intra-arterial (IA) administration of rt-PA is possible up to 8 hours after the onset of stroke.^6^^,^^7^ In several studies, it has been observed that IA thrombolysis increases recanalization rapidly as compared to IV thrombolysis.^8^ Our aim is to investigate the outcome of IA thrombolysis as a mode of treatment in cases of acute ischemic stroke within 6.5 hours of the onset of stroke.

## Materials and Methods

This prospective study was carried out between January 2008 and December 2013 at Yashoda Hospital, in Hyderabad, in the state of Telangana in South India. The study population comprised of 10 consecutive patients with acute ischemic stroke. All the patients were recruited within 6.5 hours of the onset of stroke. The World Health Organization defined stroke as rapidly developing clinical signs of focal/global disturbance of cerebral function, with symptoms lasting 24 hours or longer or leading to death, with no apparent cause other than of vascular origin.^9^

Patients who presented with acute ischemic stroke were included in the study if they were admitted in stroke unit with onset of stroke from 4.5 hours to 6.5 hours, with angiographic evidence of intravascular clots in the cerebral arteries by computerized tomography (CT) angiogram or magnetic resonance imaging (MRI) brain angiogram before initiation of therapy, and with National Institutes of Health Stroke Scale (NIHSS) score from 4 to 25.

Patients were excluded from the study if they had intracranial hemorrhage or subarachnoid hemorrhage, more than 6.5 hours after onset of stroke, rapidly improving symptoms, history of arterial puncture at a noncompressible site or lumbar puncture within 7 days, blood pressure > 200 mmHg systolic or > 120 mmHg diastolic, NIHSS score below 4 or more than 25, age more than 80 years, fibrinogen < 120 mg, below 100,000 platelet count, serum glucose levels < 50 mg/dl or > 400 mg/dl, use of anticoagulant in spite of international normalized ratio (INR), current taking oral anticoagulants with prothrombin time (PT) > 15 sec or INR > 1.7, gastrointestinal hemorrhage within 21 days, pericarditis, vasculitis, renal failure peritoneal or hemodialysis, or dementia, history of recent seizures, history of trauma or cardiopulmonary resuscitation or surgery within two weeks, active internal bleeding, pregnancy or delivery within two weeks, and genitourinary or gastrointestinal hemorrhage below 21 days.

**Figure 1:**
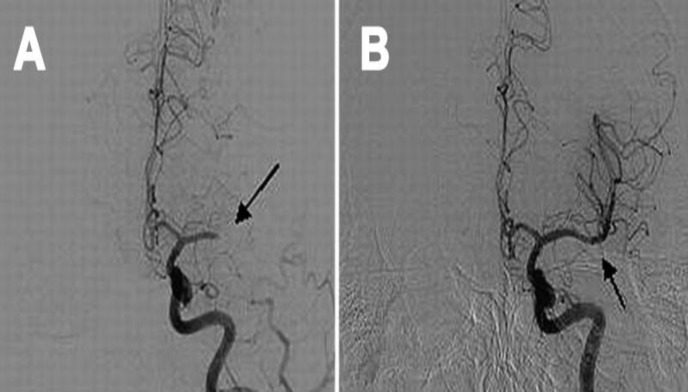
A: Grade 0: No Perfusion, and B: Complete perfusion of left middle cerebral artery (MCA) occlusion followed by reperfusion of recombinant tissue plasminogen activator (rtPA)

All stroke patients underwent a CT scan of the brain to exclude hemorrhagic stroke (Figure 1). MRI and CT angiogram (CTA) or MR angiography (MRA) of the brain were performed. A cardiac evaluation of all the patients was done. Additional tests were performed as required. The stroke specialist reviewed the data and sub-classified the strokes as large artery atherosclerosis, cardioembolic stroke, small artery disease, stroke of other determined etiology, and stroke of indeterminate etiology.^10^

Data on all the patients with acute ischemic stroke was collected from their medical records. This included demographic data, evaluation and treatment time, admission and 24 hours NIHSS scores, and time at which onset of symptoms occurred. All the patients were treated with IA rt-PA. The micro-guide catheter (size 5 F) was used to interrupt the clot. After identification of the microcatheter tip (tip length 3 mm) location, 15-20 mg of rt-PA was injected. The onset-to-CT scan/MRI of the brain, and onset-to-recanalization times were noted. 

Major neurological improvement was defined as an NIHSS score equal to 0 or 1 at 24 hours or an improvement of ≥ 8 points compared to the baseline.^11^ Complications of rt-PA treatment were assessed after infusion and follow-up. All the patients were given 20 mg of rt-PA except 2 patients have received lower dosage (15 mg). 

**Figure 2:**
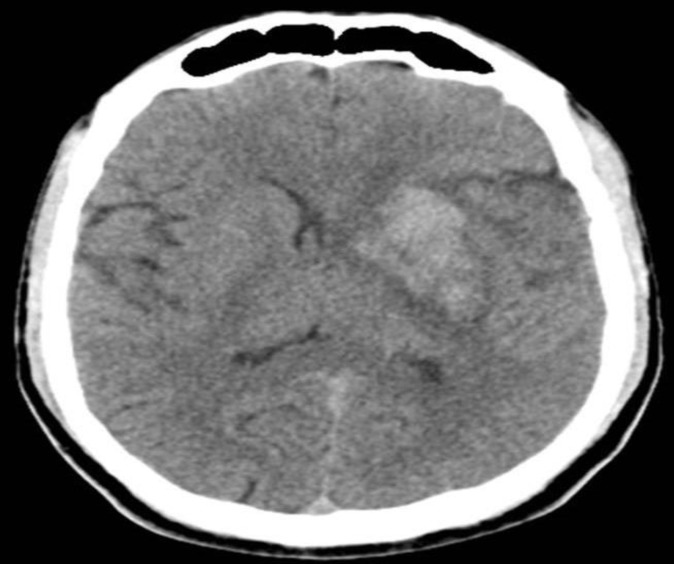
Post thrombolysis CT scan of brain shows hemorrhagic transformation

We assessed all patients by Thrombolysis in Cerebral Infarction (TICI) score after treatment: no perfusion, score 0; minimal perfusion, score 1; partial perfusion, score 2; only partial filling (less than two-thirds), score 2a; complete filling in all arteries but the filling is slower than normal, score 2b; and complete perfusion, score 3 (Figure 2).^12^

All patients were followed-up on a regular basis for assessment of outcomes at one week, 30, 60, and 90 days. All patients were also assessed with NIHSS score at baseline, one week, 30, 60, and 90 days. Barthel Index (BI) and Modified Rankin Scale (mRS) score were measured at one week, 30, 60, and 90 days. The predictive value of major neurological improvement at 90 days was analyzed using the mRS score and a good outcome was defined as a mRS score of ≤ 2 at 90 days.^4^

The study protocol was approved by the Institutional Ethics Committee (IEC) and informed consent was obtained from all the participants. If patients were unable to sign the consent forms due to hemiplegia, consent was obtained from the patients’ relatives.

Univariate comparisons were drawn where NIHSS scores were recorded and compared at the time of admission, and thereafter at 24 hours and 90 days using the Student’s t-test. Similar analysis was performed for BI and mRS scores compared between week 1 and 90 days after stroke.

## Results

Seventeen hundred patients presenting with ischemic stroke were evaluated during the study period, 72 (4.2%) patients received IV rt-PA and 10 (0.6%) patients were treated with IA rt-PA. The study population comprised nine men (90%) and one woman (10%). The mean age was 56.3 ± 1.8 years. The prevalence of risk factors among the study population was as follows: 6 (60%) patients had hypertension, 5 (50%) had diabetes mellitus, and 4 (40%) had hyperlipidemia. Out of 10 patients, occlusion was observed in the middle cerebral artery (MCA) in 6 (60%) patients. One patient each had occlusions in the anterior cerebral artery (ACA), posterior cerebral artery (PCA), basilar artery, and T junction (Table 1). 

The NIHSS scores was measured at the time of admission, after 24 hours and at 90 days of follow-up (Table 2). 

There was a significant improvement in the NIHSS score at 24 hours (6.8 ± 2.2) and at the 90 days follow-up (1.3 ± 1.4) as compared to the score at the time of admission (15.3 ± 1.3) (P < 0.0001). 

Mean BI (97.5 ± 2.6) and mRS (1.5 ± 1.1) scores were significantly improvement at 90 days follow-up compared to BI (75.5 ± 10.0) and mRS (2.5 ± 1.9) (P < 0.0010) scores at 1 week follow-up.

**Table 1:** Baseline characteristics

**Parameters**	**Number ** **(n = 10)**
Men [n (%)]	9 (90)
Mean age (year) (mean ± SD)	56.3 ± 1.8
Age range (years)	35-68
MCA occlusion [n (%)]	6 (60)
ACA occlusion [n (%)]	1 (10)
PCA occlusion [n (%)]	1 (10)
T Junction occlusion [n (%)]	1 (10)
Basilar artery [n (%)]	1 (10)
Hemorrhagic transformation [n (%)]	1 (10)
Hypertension [n (%)]	5 (50)
Diabetes mellitus [n (%)]	4 (40)
Hyperlipidemia [n (%)]	3 (30)
Smoker [n (%)]	3 (30)
Alcoholics [n (%)]	3 (30)
Onset of stroke range (hours)	3-6
Mean time for opening the artery (hours) (mean ± SD)	5.6 ± 0.4
Mean dose of rt-PA (mg)	18.7
Stroke subtyping	
Large artery atherosclerosis [n (%)]	6 (60)
Stroke of indeterminate etiology [n (%)]	3 (30)
Stroke of other determinate etiology [n (%)]	1 (10)

MCA: Middle cerebral artery; ACA: Anterior cerebral artery; PCA: Posterior cerebral artery; rt-PA: Recombinant tissue plasminogen activator; SD: Standard deviation

**Table 2:** Clinical data on the National Institutes of Health Stroke Scale score at admission and at the 90 days follow-up

**Serial ** **number **	**Site of ** **hemiplegia**	**Site of ** **occlusion**	**Time of ** **recanalization ** **after onset of ** **stroke (hours)**	**Dosage ** **of ** **rt-PA ** **(mg)**	**mTICI ** **score**	**NIHSS ** **score on ** **admission**	**NIHSS ** **score ** **at 24 ** **hours**	**NIHSS ** **score ** **at 90 ** **days**	**mRS ** **score ** **at 90 ** **days**
1	Left side	MCA	5.5	20	3	16	8	0	0
2	Right side	PCA	5.5	15	2a	18	12	5	3
3	Right side	MCA	6.0	20	2a	15	8	5	1
4	Left side	T Junction	6.0	20	3	17	7	1	1
5	Right side	MCA	6.0	15	3	14	6	0	0
6	Left side	ACA	5.0	20	3	15	4	0	0
7	Right side	MCA	6.0	20	2a	15	10	7	2
8	Left side	MCA	5.0	20	3	14	6	1	1
9	Right side	Basilar artery	5.5	20	3	15	8	6	3
10	Left side	MCA	6.0	20	3	14	5	Lost to follow- up	Lost to follow- up

SL No: MCA: Middle cerebral artery; ACA: Anterior cerebral artery; PCA: Posterior cerebral artery; rt-PA: Recombinant tissue plasminogen activator; NIHSS: National Institutes of Health Stroke Scale

## Discussion

The IA thrombolysis is a universally accepted treatment for acute ischemic stroke. We found in our study that 70% of our patients had good outcome at 90 days follow-up. Similar findings were noted by Huded et al. (47%)^13^ and Wonget al. (38%).^14^ Lisboa et al.^15^ analyzed and found that in patients with acute ischemic stroke, outcome was significantly better in patients who underwent IA thrombolysis with low mortality rate compared to control group. 

Most of our study patients had IA clots in MCA (6/10) with the remaining few having clots in ACA (1/10) T Junction (1/10), BA (1/10) and PCA (1/10). The IA thrombolysis has been shown to be useful and comparatively safe mode of treatment for selected patients with anterior, middle, and posterior circulation strokes.^16^ However, in our study poor outcome was noticed in posterior circulation stroke.

In our study, good recanalization with 70% of TICI grade 3 and 30% of TICI grade 2a was achieved after IA thrombolysis, which were advocated by others.^17^^-^^26^ In our study, we used only IA rt-PA in patients who were not eligible for IV thrombolysis. Many studies have used a combination of IV thrombolysis and IA thrombolysis.^21^

Huded et al. also evaluated the outcome of isolated IA thrombolysis with urokinase or tpa in their study and showed that 53% had TIMI 2 or 3 recanalization.^13^ Mattle et al.^21^ compared IV with IA thrombolysis and noted in their study a recanalization grade of TIMI 2 or 3 in 71% after IA thrombolysis and twofold increase of the recanalization with IA compared to IV thrombolysis. 

Shaltoni et al.^18^ showed that recanalization with TICI flow > 2a occurred in 73% of patients who received IV thrombolysis followed by IA thrombolysis with one of the three urokinase, reteplase, or alteplase. Tomsick et al.^19^ in IMS-II demonstrated a TICI 2/3 in 61.3% while a partial or complete recanalization occurred in 74.6% of patients who underwent IV thrombolysis with IA thrombolysis and low energy sonography.

Park et al.^25^ studied a combination of early IV thrombolysis followed by IA thrombolysis and showed a recanalization rate after inhospital thrombolysis of 65.2% and outside hospital thrombolysis of 70%.

Qureshi et al.^26^ combined treatment on IA reteplase with intravenous abciximab for acute ischemic stroke and demonstrated a recanalization rate of 65%. Heo et al.^23^ noted in their study on IA thrombolysis or thrombolytics with or without mechanical treatments, recanalization rate was 60% to 80%.^23^

Although the effectiveness of IA thrombolysis has been demonstrated in randomized clinical trials, several issues about the usage are yet to be clarified. In the present study, we tried to identify the efficacy of IA rt-PA in the treatment of acute ischemic stroke in Indian population. The IA rt-PA advantages are the higher concentration of medication that directly enters into occluding arteries and less complications of hemorrhagic transformation.^26^ All these may lead to a higher recanalization rate as demonstrated by the present study. Further, due to the longer therapeutic window for IA thrombolysis, more stroke patients may benefit in comparison to IV thrombolysis. 

***Hemorrhagic transformation***

In our study we found hemorrhagic transformation in 1 (10%) patient. Studies have established prevalence of hemorrhagic transformation from 4.7% to 25%.^27^^,^^28^ Lisboa et al.^15^ found that intracerebral haemorrhage was significantly higher with IA treatment (9.5%). Shaltoni et al.^18^ found a prevalence of intracerebral hemorrhage in 5.8% of patients. Mattle et al.^21^ noted that one patient had intracerebral hemorrhage.^22^ However, Yoon et al.^24^ found no symptomatic hemorrhage in their study.

***Outcome***

In the present study, we found significant improvement after IA thrombolysis in our patients. The NIHSS score decreased significantly after treatment with IA thrombolysis as advocated by other studies.^28^ At 90 days follow-up, good outcome (mRS score ≤ 2) was seen in 7 patients (70%) and poor outcome was seen in 2 patients. A similar study showed at 90 days follow-up an mRS ≤ 2 in 9 and poor outcome (mRS: 3-5) in 3 patients.^25^

Huded et al.^13^ showed that 47.1% of patients after IA thrombolysis had mRS score of less than or equal to 2 at 90 days follow up. Lisboa et al.^15^ noted that good outcome was significantly higher (41.5%) in IA thrombolysis group compared to control group (23%). Mattle et al.^21^ showed in their study that good outcome was significantly increased in IA thrombolysis group (53%) compared to IV thrombolysis group (23%) (P = 0.0200). Nam et al.^29^ demonstrated an increase in good outcome by 20% after IA thrombolysis compared to IV thrombolysis. Some studies established IA thrombolysis as more advantageous and independently associated with good outcome,^21^ however some studies found poor functional outcomes.^30^

In our study we found two patients with poor outcome (mRS > 3), both had posterior circulation strokes with complete basilar artery occlusion in one and PCA occlusion in the other patient. Macleod et al.^31^ noted a good outcome of 50% in their study on IA rterial urokinase in posterior circulation stroke within 24 hours. Voetsch et al.^32^ noted 59% with basilar artery occlusion had good outcome. A lower percentage of good outcome (17-40%) was seen by Power^33^ after IA thrombolysis in basilar artery occlusion and there was no difference if the treatment was given < 6 hours or > 6 hours after the onset of basilar artery occlusion. 

The IA thrombectomy in the posterior circulation is less favourable as it is strongly associated with the futile recanalization. Futile recanalisation is defined as poor outcome even after good recanalisation and may be related to the age of the patient, size of the core infarct and presence of collaterals. Hence, future studies are required in posterior circulation to clarify who will benefit from IA thrombectomy.^34^

***Mortality***

In our study all our subjects were alive at 90 days follow-up with no mortality, similar to previous studies.^26^ Our sample size was small. The mortality rates in other studies are varied and probably depended on multiple factors such as disease severity, time of IA thrombolysis, other comorbidities and duration of hospital stay.^14^^,^^22^^,^^35^^,^^36^ Ogawa et al.^35^ noted a slightly higher mortality rate of 5.3% in patients who underwent IA thrombolysis with urokinase, compared with control group (3.5%) at 90 day follow-up. Mattle et al.^21^ on the other hand, showed a significantly lower mortality rate IA thrombolysis group (7%) compared to IV thrombolysis group (23%) (P = 0.0200). A much higher mortality rate 33% was documented by Natarajan et al.^36^ in patients who received IA thrombolysis with other endovascular treatments within 8 hours of onset of stroke at 90 days follow up. 

The disadvantages of IA thrombolysis include additional delay because an angiography has to be performed and a microcatheter needs to be placed before commencement of therapy and additional risks associated with the endovascular procedure. With the advent of stent retrievers, studies suggest that IA rt-PA may be less effective compared to the second generation mechanical thrombectomy devices.^37^

However, the stent retrievers are very expensive and can be used only in a few patients in a country like India. On the other hand, IA rt-PA is a reasonably priced easy and efficient method to treat cases of acute ischemic stroke and our experience suggests that the benefit can be reaped by many. 

In our study limitations include the small sample size as it was based on a small, single center involvement, non-randomized design and non-blinded nature of the study without a control group. The strength of our study was that it is a prospective study and recanalisation analysis was done by TICI score with a 90% follow-up rate.

## Conclusion

Our study has demonstrated that IA thrombolytic therapy has significant benefit even up to 6.5 hours onset of stroke and might be beneficial in this selected population who are not fit for IV rt-PA. However, multicentric and large scale studies are required to confirm this benefit. The benefits also suggest that a combination therapy of IV thrombolysis and IA thrombolysis may help in our population within the specified time window.

## Notes:

**How to cite this article: **Chaudhuri JR, Kumar R, Umamahesh M, Rukmini Mridula K, Alladi S, Bandaru S. Outcome of acute ischemic stroke after intra-arterial thrombolysis: A study from India. Iran J Neurol 2016; 15(4): 195-201.
